# Tumour necrosis factor inhibitors in inflammatory bowel disease: the story continues

**DOI:** 10.1177/17562848211059954

**Published:** 2021-12-09

**Authors:** Laurent Peyrin-Biroulet, William J. Sandborn, Remo Panaccione, Eugeni Domènech, Lieven Pouillon, Britta Siegmund, Silvio Danese, Subrata Ghosh

**Affiliations:** Department of Gastroenterology and Inserm NGERE U1256, University Hospital of Nancy, University of Lorraine, Vandoeuvre-lès-Nancy, France; University of California, San Diego, La Jolla, CA, USA; Cumming School of Medicine, University of Calgary, Calgary, AB, Canada; Inflammatory Bowel Disease Unit, University of Calgary, Calgary, AB, Canada; Hospital Universitari Germans Trias i Pujol, Badalona, Spain; Departament de Medicina, Universitat Autònoma de Barcelona, Barcelona, Spain; Centro de Investigación Biomédica en Red sobre enfermedades Hepáticas y Digestivas CIBEREHD, Spain; Imelda GI Clinical Research Centre, Imeldaziekenhuis Bonheiden, Bonheiden, Belgium; Medizinische Klinik für Gastroenterologie, Infektiologie und Rheumatologie, Charité – Universitätsmedizin Berlin, corporate member of Freie Universität Berlin, Humboldt-Universität zu Berlin and Berlin Institute of Health, Berlin, Germany; Gastroenterology and Endoscopy, IRCCS Ospedale San Raffaele, Milan, Italy; University Vita-Salute San Raffaele, Milan, Italy; Institute of Immunology and Immunotherapy, University of Birmingham, Birmingham, B15 2TT, UK; NIHR Biomedical Research Centre, University of Birmingham and Queen Elizabeth Hospital Birmingham, Birmingham, B15 2TH, UK

**Keywords:** Crohn’s disease, TNF inhibitors, ulcerative colitis

## Abstract

In the 1990s, tumour necrosis factor-α inhibitor therapy ushered in the biologic therapy era for inflammatory bowel disease, leading to marked improvements in treatment options and patient outcomes. There are currently four tumour necrosis factor-α inhibitors approved as treatments for ulcerative colitis and/or Crohn’s disease: infliximab, adalimumab, golimumab and certolizumab pegol. Despite the clear benefits of tumour necrosis factor-α inhibitors, a subset of patients with inflammatory bowel disease either do not respond, experience a loss of response after initial clinical improvement or report intolerance to anti-tumour necrosis factor-α therapy. Optimizing outcomes of these agents may be achieved through earlier intervention, the use of therapeutic drug monitoring and thoughtful switching within class. To complement these approaches, evolving predictive biomarkers may help inform and optimize clinical decision making by identifying patients who might potentially benefit from an alternative treatment strategy. This review will focus on the current use of tumour necrosis factor-α inhibitors in inflammatory bowel disease and the application of personalized medicine to improve future outcomes for all patients.

## Introduction

Inflammatory bowel diseases (IBD), such as ulcerative colitis (UC) and Crohn’s disease (CD), affect millions of people worldwide and exert a significant burden on patients and health care providers.^1-3^

**Figure 1.**
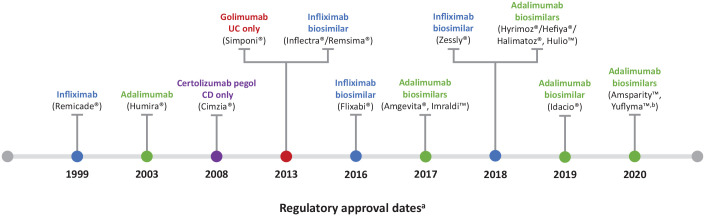
Currently available TNF inhibitors approved for the treatment of IBD. Data extracted from previous studies.^11-28^ ^a^All dates are based on EU approvals, apart from certolizumab pegol, which is approved in the United States only. ^b^Pending European Commission approval; the Committee for Medicinal Products for Human Use has adopted a positive opinion for the granting of marketing authorization. CD, Crohn’s disease; EU, European Union; IBD, inflammatory bowel disease; TNF, tumour necrosis factor; UC, ulcerative colitis.

The introduction of monoclonal antibodies targeting tumour necrosis factor-α (TNF) in the late 1990s was a major breakthrough in the treatment of IBD and led to significantly improved outcomes, including prolonged clinical remission, prevention of complications and restoration of patients’ quality of life.^4-7^ The first TNF inhibitor to be developed for IBD was infliximab.^5^ Subsequently, three other TNF inhibitors – adalimumab, golimumab and certolizumab pegol – have been approved as treatments for UC and/or CD.^8^ In contrast, etanercept^9^ and onercept were not effective.^10^ Since 2013, a number of biosimilars to infliximab and adalimumab have been approved for the treatment of IBD. A summary of currently available TNF inhibitors is shown in Figure 1.^11-28^

Despite the clear benefits of TNF inhibitors, a subset of patients with IBD experience primary non-response to therapy or secondary loss of response.^29^ Optimizing the use of TNF inhibitors through earlier intervention, therapeutic drug monitoring (TDM) or switching anti-TNF agents may improve outcomes.^30,31^ Furthermore, use of evolving predictive biomarkers may enable earlier identification of patients who would potentially benefit from an alternative treatment strategy.^32^

The objective of this narrative review is to present a state-of-the-art summary of the use of TNF inhibitors in IBD. The review focusses on recent data and the use of personalized medicine to improve outcomes for all patients. The safety of TNF inhibitors was extensively reviewed by Shivaji *et al.*^33^ in 2019 and is therefore not the focus of this review.

## Literature search

A literature search was conducted in May 2020. The PubMed database was searched using terms relating to disease areas, TNF inhibitors, early treatment, dose optimization, TDM, biomarkers, loss of response, treatment failure and biosimilars. The search strings are provided in Supplementary Table 1. As TNF inhibitors have been extensively reviewed previously, only articles in English published from January 2018 were included, to ensure a focus on the most recent literature. Additional key references were identified through searching the bibliographies of retrieved articles.

## Role of TNF in IBD

TNF is a 17 kDa soluble cytokine that is secreted predominantly by monocytes and can exert potent proinflammatory effects on a number of different cell types.^34^ It plays an important role in intestinal homeostasis (reviewed in detail^8^ and references therein) and is involved in multiple physiological processes, including regulation of epithelial cell shedding during renewal of the intestinal epithelium, maintenance of epithelial barrier integrity, wound healing and mucosal repair. Conversely to its function in gut health, TNF also plays a pivotal role in the development and perpetuation of IBD, as illustrated by the clinical benefits associated with TNF inhibition.^8^

The role of TNF in the pathogenesis of IBD has been reviewed previously in detail.^8,34,35^ In brief, both soluble and membrane-bound TNF, along with other proinflammatory cytokines such as interleukin (IL)-1β, IL-6 and IL-18, are produced by a variety of stromal and immune cells within the inflamed mucosa. Non-immune cells also produce proinflammatory cytokines; for example, members of the IL-1 cytokine family, including IL-18, are produced by intestinal epithelial cells, while TNF and IL-6 are produced by stromal fibroblasts. Through its receptors, TNFR1 and TNFR2, TNF exerts pleiotropic proinflammatory effects, including angiogenesis, induction of Paneth cell death, production of matrix metalloproteases, and activation of macrophages and effector T cells. Experiments in mice have demonstrated that membrane-bound, rather than soluble, TNF may play a significant role in intestinal inflammation.^36,37^ Consistent with this, clinically effective antibodies such as infliximab, adalimumab and certolizumab pegol that neutralize both soluble and membrane-bound forms of TNF^38-40^ have been shown to induce mainly CD4+ T-cell apoptosis *in vivo*.^41^ These inhibitors have also been shown to specifically affect CD4+ and CD8+ T cells, by promoting and maintaining an anti-inflammatory IL-10+ phenotype and delaying CD4+ T-cell activation, maturation and proliferation.^42-44^ In addition, infliximab and adalimumab, but not certolizumab, have been shown to induce wound-healing macrophages *in vitro* and *in vivo via* an Fc-receptor-mediated mechanism.^5,45^ In contrast, etanercept, which predominantly blocks soluble TNF, has been shown to have no therapeutic effect in IBD when used at doses that have been approved for the treatment of other autoimmune diseases.^9,41,46^

TNF may also play a role in extraintestinal manifestations (EIMs), which have been reported in up to 47% of patients with IBD.^47^ The musculoskeletal system is the most frequently affected, with peripheral arthritis and axial spondyloarthritis reported as the most common manifestations; other organs affected include the skin, bile duct and eyes.^47^ The exact pathogenic mechanisms relating to the development of EIM remain to be fully defined. However, TNF has been implicated in EIM development; for example, TNF has been shown to be upregulated in skin biopsies taken from patients with cutaneous EIMs.^48^ Furthermore, results from a systematic review indicated that adalimumab and infliximab can be effective treatments for musculoskeletal, cutaneous and ocular EIMs.^49^ In addition to IBD, aberrant TNF signalling underlies many other chronic inflammatory conditions, including psoriasis, psoriatic arthritis, juvenile idiopathic arthritis, rheumatoid arthritis and ankylosing spondylitis.^50,51^

## Current position of TNF inhibitors and challenges for treatment guidelines

Current treatment guidelines on the use of TNF inhibitors are summarized in Table 1 for UC^52-56^ and Table 2 for CD.^57-61^ The guidelines generally note the low quality of the evidence available to guide optimal use of TNF inhibitors in clinical practice, and this can lead to discrepancies between the guidelines. For example, the European Crohn’s and Colitis Organisation (ECCO) highlights that early treatment (within the first 2 years of the disease) can potentially benefit patients with CD, but note that this remains a matter of debate as results are based on *post hoc* analyses of clinical trials.^57^ Recent UC guidelines include recommendations on early intervention or the use of combination therapy but are careful to describe the evidence as low quality due to a lack of studies. Despite inclusion in IBD guidelines, uncertainties remain about the benefit and timing of combination therapy. In an American Gastroenterological Association (AGA) clinical practice update, Hanauer *et al.* advised that the addition of thiopurines or methotrexate may reduce immunogenicity and increase trough levels of TNF inhibitors,62 although the advantages of combination therapies must be considered in the context of additional safety concerns, including potentially increased risks of opportunistic infections and certain types of lymphoma.^62-64^ For patients who have achieved remission, discontinuing immunosuppressants may offer safety benefits, but this needs to be balanced against the risks of relapse and bowel damage.^65^ The benefits of de-escalation were initially reported in a small, open-label study in CD, which demonstrated that continuation of immunosuppression beyond 6 months provided no clinical benefit over infliximab monotherapy.^66^ However, this study had several methodological limitations, and it is difficult to conclude from these data alone that thiopurines can be safely discontinued after 6 months of treatment. The same group reported an association between trough infliximab levels >5 µg/mL at the time of immunomodulator withdrawal and a decreased risk for subsequent loss of response to infliximab.^67^ Yet, in this retrospective study, patients received combination therapy for a median of 13 months before withdrawal, indicating that the patient population was potentially biased towards those with a low likelihood of TNF immunogenicity. A more recent retrospective study in children with CD demonstrated that stepping down from combination therapy to TNF inhibitor monotherapy was associated with a higher risk of disease exacerbation, hospital admission and surgery.^68^ Dose reduction of the immunomodulator may also be considered. A prospective, open-label, randomized trial in patients with a durable remission (⩾6 months) under combination therapy demonstrated that halving the azathioprine dose was as effective as continuation at full dose; however, this study was uncontrolled and underpowered, and therefore results must be interpreted with caution.^69^ A recent Cochrane review confirmed that the evidence supporting de-escalation of immunosuppressant therapy in IBD was of low quality and that high-quality randomized controlled trials are required.^65^ An alternative to withdrawal or dose reduction of the immunomodulator may be de-escalation of the TNF inhibitor by increasing the interval between doses. Limited evidence is emerging to suggest that up to two-thirds of patients with IBD could potentially maintain remission while reducing frequency of infliximab or adalimumab dosing,^70-73^ and a reduction in TNF-inhibitor-related adverse events has been reported with adalimumab.^72^ C-reactive protein (CRP) levels and disease duration appear to be indicative of patients who may have success with this approach.^71,73^

**Table 1.** Current guidelines for the use of TNF inhibitors in UC.

ECCO^52^	ACG^53^	AGA^54^	ECCO/ESPGHAN^55,56^
● Induction and maintenance of remission in adults with moderate-to-severe active disease that is refractory to conventional medications^a^ or steroid-dependent, or for those hospitalized with IV steroid-refractory acute severe UC ● Combine with thiopurines in patients with steroid-dependent disease, moderate oral steroid-refractory disease or moderate colitis refractory to thiopurines	● Combine with thiopurines for induction therapy	● Early intervention for adults with moderate-to-severe UC (with or without an immunomodulator) rather than a step-up approach^b^ ● Combine with thiopurines or MTX rather than thiopurine monotherapy in adults with moderate-to-severe UC^b^	● Induce and maintain remission in chronically active UC or refractory UC and in children hospitalized with IV steroid-refractory acute severe UC ● Combine infliximab with thiopurines to reduce immunogenicity of infliximab and to enhance effectiveness; discontinuation of thiopurines can be considered after 6 months, especially in boys and preferably after ensuring trough infliximab levels of ⩾5 µg/mL^c^

5-ASA, 5-aminosalicylic acid; ACG, American College of Gastroenterology; AGA, American Gastroenterological Association; ECCO, European Crohn’s and Colitis Organisation; ESPGHAN, European Society of Paediatric Gastroenterology, Hepatology and Nutrition; IV, intravenous; MTX, methotrexate; TNF, tumour necrosis factor; UC, ulcerative colitis.

a For example, 5-ASA or immunomodulators.

b Based on low-quality evidence due to a lack of randomized, controlled studies.

c ECCO/ESPGHAN guidelines state that thiopurine monotherapy does not have a favourable benefit–risk ratio, and the value of combining thiopurines with TNF inhibitors other than infliximab in paediatric patients is more controversial.^55^

**Table 2.** Current guidelines for the use of TNF inhibitors in CD.

ECCO^57^	ACG^58^	AGA^59^	ECCO/ESPGHAN^60^
● Induce and maintain remission in patients with moderate-to-severe CD refractory to other treatments ● Combine with thiopurines in adults with moderate-to-severe CD to induce remission	● Induce and maintain remission in patients with moderate-to-severe CD refractory to other treatments, severely active CD and in those with perianal fistulizing disease ● After surgical resection, combine with thiopurines as first-line prophylactic treatment over endoscopy-guided treatment (very low-quality evidence) ● Initiation or optimization of combination therapy with thiopurines over monitoring alone in those with asymptomatic endoscopic recurrence (moderate-quality evidence^a,61^)	● Induce and maintain remission in patients with moderate-to-severe CD refractory to other treatments ● Combine with thiopurines in adults with moderate-to-severe CD to induce remission	● Induce remission in new-onset patients with a high risk for a complicated disease course ● Induce and maintain remission in children with immunomodulator-refractory active CD ● Consider upfront use in combination with an immunomodulator in patients with perianal disease, stricturing or penetrating behaviour, or severe growth retardation

ACG, American College of Gastroenterology; AGA, American Gastroenterological Association; CD, Crohn’s disease; ECCO, European Crohn’s and Colitis Organisation; ESPGHAN, European Society of Paediatric Gastroenterology, Hepatology and Nutrition; TNF tumour necrosis factor.

a The guidance was based on indirect evidence from the AGA clinical guidelines on the role of anti-TNF and immunomodulators in the maintenance of remission in patients with inflammatory luminal CD, and the authors acknowledge that thiopurine monotherapy may have potentially lower efficacy.^59,61^

## Earlier intervention with TNF inhibitors

The benefits of early intervention with TNF inhibitors in CD have been reported in a recent meta-analysis of real-world and prospective clinical trial data, which demonstrated that biologic treatment within 2 years of diagnosis was associated with improved clinical outcomes (clinical remission, corticosteroid-free clinical remission, mucosal healing, relapse rate, hospitalization rate, complications and surgeries) compared with late/conventional management in adult and paediatric patients.^74^ Furthermore, a *post hoc* analysis of the CHARM (Crohn’s Trial of the Fully Human Antibody Adalimumab for Remission Maintenance) and follow-on open-label extension Additional Long-Term Dosing With HUMIRA to Evaluate Sustained Remission and Efficacy in CD (ADHERE) trial demonstrated increased clinical remission rates when adalimumab treatment was initiated early in the disease course.^75^ Moreover, data from the prospective EXTEND (Extend the Safety and Efficacy of Adalimumab through Endoscopic Healing) study demonstrated that 33% of patients with a disease duration ⩽2 years achieved deep remission – defined as the absence of mucosal ulceration plus clinical remission (Crohn’s Disease Activity Index <150) – following 52 weeks of continuous adalimumab treatment, compared with 20% and 16% of patients with disease duration of >2–5 and >5 years, respectively.^76^

Many patients with CD develop bowel damage in the long term,^77^ and several studies have evaluated whether the timely use of TNF inhibitors can prevent or delay this. A prospective evaluation of patients with CD showed that early introduction of TNF inhibitor therapy was associated with reduced cumulative bowel damage – assessed using the Lémann Index (LI).^78^ In a validation study,^79^ the low LI in the first 2 years suggests a possible window of opportunity within which intensive treatment might delay or prevent disease progression. Furthermore, in a recent retrospective longitudinal study in adult patients, the use of TNF inhibitors within 3 months of diagnosis correlated with a slower rate of bowel damage (non-significant trend), reflected by the reduced rate of progression of LI (*p* = 0.069).^80^ Following a multivariable logistic regression analysis to adjust for potential confounding, the association between LI and earlier initiation of biologics was significant (*p* = 0.03) and showed that later intervention with TNF inhibitors decreased the odds of disease regression or stabilization by 91%. Similarly, in a paediatric study, fewer patients with CD who received TNF inhibitors within 3 months of diagnosis moved from an inflammatory phenotype to stricturing or penetrating disease compared with those who did not receive early treatment.^81^ Conversely, an earlier paediatric inception, prospective, cohort study observed that early intervention with TNF inhibitors was only significantly effective at reducing the risk of penetrating complications and not stricturing complications.^82^ These studies highlight the possibility of a therapeutic window in which the course of the disease can be modified. However, the results must be interpreted with caution as the studies performed to date have a number of limitations, including the use of surrogate endpoints and a lack of long-term data on the impact of early treatment with TNF inhibitors. In addition, many patients with CD have a more indolent disease course and do not develop complications.^83,84^ A personalized approach rather than universal early treatment with TNF inhibitors may therefore be needed to identify patients at risk of structural damage.

Given the potential impact of early treatment, some patients may benefit from a top-down or accelerated step-up treatment strategy rather than a more conventional approach with the incremental use of therapies. The 2-year, open-label, randomized Step-up/Top-down trial demonstrated that early intervention with top-down therapy was superior to conventional step-up management in patients with CD who had not previously received treatment with corticosteroids, antimetabolites or biologics. Top-down treatment consisted of three infusions of infliximab (weeks 0, 2 and 6) combined with long-term maintenance treatment with azathioprine; additional treatment with infliximab was administered if necessary.^85^ A retrospective review of patients with long-term follow-up (median of 8 years) demonstrated that clinical and endoscopic remission rates were similar for step-up and top-down strategies.^86^ However, top-down treatment was associated with lower relapse rates and longer time to relapse compared with conventional treatment. This group was also less frequently treated with corticosteroids and TNF inhibitors. Furthermore, mucosal healing 2 years after the start of treatment was associated with a reduced use of TNF inhibitors during long-term follow-up.^86^ Similarly, the SONIC (Study of Biologic and Immunomodulator Naive Patients in Crohn’s Disease) trial demonstrated that, in patients naïve to immunomodulators and biologics, top-down therapy (infliximab infusions at weeks 0, 2, 6, 14 and 22 combined with daily azathioprine) was more effective than azathioprine alone.^87^ An accelerated step-up approach in a community setting was investigated in the REACT (Randomized Evaluation of an Algorithm for Crohn’s Treatment) trial. This study demonstrated that early combined immunosuppression (ECI) with a TNF inhibitor and antimetabolite did not significantly improve clinical remission rates compared with conventional management. However, the risk of major adverse outcomes (surgery, hospital admission or serious disease-related complications) was lower in the ECI group, suggesting the natural history of CD can potentially be altered following early initiation of effective therapy.^88^ Of note, the lack of impact on clinical remission rates in the overall population raises the possibility that a more personalized approach through the use of predictive biomarkers may be required to identify specific patients who would likely benefit from different treatment strategies.^89^ More recently, the open-label, phase III CALM trial demonstrated that employing a treatment algorithm to monitor inflammatory activity and clinical symptoms (tight control) led to improved clinical and endoscopic outcomes in patients with early CD compared with an algorithm based on clinical symptom monitoring alone.^90^ In this study, both groups (patients managed with tight control and patients managed with a clinical management algorithm) received stepwise treatment escalation with increased clinical symptoms; however, in the tight control group, treatment escalation was also initiated when certain inflammatory biomarker levels were reached – CRP ⩾5 mg/L or faecal calprotectin ⩾250 µg/g.

Other recent prospective and retrospective studies that support early intervention with TNF inhibitors in adult and paediatric patients with CD are summarized in Supplementary Table 2 Overall, although the data on early intervention are promising, results must be interpreted with caution due to limitations associated with studies performed to date. This is emphasized by the ECCO guidelines, which note that TNF inhibitors may be more effective if introduced earlier in the disease course, but with the caveat that results are based on *post hoc* analyses from clinical trials.^57^

There are currently limited data on the efficacy of early intervention with TNF inhibitors in UC, and results suggest that this strategy may offer limited benefits to this patient population. Yet, it is difficult to draw conclusions, as most of the study populations are small.^91,92^ Despite the ‘very low’ quality of this evidence, the AGA recommends the early use of TNF inhibitors for adults with moderate-to-severe UC (with or without an immunomodulator) rather than a step-up approach.^54^ The Canadian Association of Gastroenterology also currently suggests early use of TNF inhibitors if patients fail to respond to steroid treatment within 2 weeks of therapy.^93^ Further long-term, large-scale studies are needed in UC to clarify whether TNF inhibitors can modify the disease course and prevent progression, especially if used early.

## Dose optimization

TDM in patients with IBD is the measurement of serum drug concentrations and anti-drug antibodies in an individual patient, to optimize dosing, inform treatment selection and maximize clinical benefits.^94,95^ Reactive TDM is used to adjust therapy in patients with a loss of response to previously successful treatment. Conversely, proactive TDM is used to reduce the risk of future disease activity or treatment failure due to subtherapeutic dosing or to reduce treatment intensity in the case of supratherapeutic dosing.^94,95^ Care must be taken when comparing reactive with proactive TDM, as they relate to different indications or populations.^94^ The ideal utilization of TDM is highly debated, as supporting evidence remains limited.^96^

Reactive TDM is superior to standardized dose optimization regimens at treatment failure and is recommended by the AGA to guide treatment changes in patients with active IBD receiving TNF inhibitors.^94,97^ The American College of Gastroenterology (ACG) guidelines also suggest reactive TDM at treatment failure,^53,58^ and ECCO–European Society of Gastrointestinal and Abdominal Radiology (ESGAR) guidelines state that reactive TDM may be beneficial in patients with IBD who are non-responsive to TNF inhibitors.^98^

The benefits of proactive TDM over standard maintenance dosing have not been convincingly demonstrated^94^ and, based on this, the AGA makes no recommendation regarding proactive TDM.^97^ Indeed, two prospective studies, the TAXIT (Trough Concentration Adapted Infliximab Treatment) and TAILORIX studies, failed to show clinical benefits of proactive TDM in patients with IBD receiving TNF inhibitors, although this may be due, at least in part, to methodological issues in these studies.^99-101^It is notable that in TAXIT, patients in the proactive TDM group experienced fewer disease flare-ups during treatment,^99^ and infliximab discontinuations occurred earlier in the clinical-based dosing group, when considering long-term outcomes.^101^ Limitations associated with the TAILORIX study methodology should also be considered; patients in the proactive TDM groups could also have been dose-optimized based on clinical symptoms alone, and there was a delay in gaining results from the central laboratory so dose calculations were based on the trough level measured before the previous infusions.^100^ Recent data from the phase III SERENE maintenance study in UC demonstrated a trend towards improved outcomes with an intensive adalimumab maintenance regimen (40 mg every week [EW]), compared with standard dosing (40 mg every other week [EOW]); however, this did not reach statistical significance after 8 weeks of maintenance treatment (in addition to the 8-week induction phase). A third group of patients was dosed according to TDM, but no benefits were reported compared with patients treated with standard or intensive adalimumab dosing.^102^ Although there was a trend towards better outcomes with the intensive regimen, it may be that the study population was too broad and not selective for patients who did not respond to standard induction dosing, dose escalation occurred too late, that the target levels were not correct or that a longer follow-up time was needed. Significant benefits of proactive TDM have been reported in paediatric patients. Children with CD who were biologic-naïve, but had responded to adalimumab induction therapy, experienced significantly higher rates of corticosteroid-free remission following proactive *versus* reactive monitoring in a non-blinded, randomized controlled trial.^103^ Despite the lack of supporting evidence, the IBD Sydney Organisation and the Australian Inflammatory Disease Working Group recommend proactive TDM (in addition to reactive TDM) based on limited observational data.^104^ In addition, a consensus statement from experts based on a modified Delphi method concluded that proactive TDM after induction and at least once during maintenance therapy was appropriate for TNF inhibitors, but not other biologics, and that reactive TDM was appropriate for primary non-responders and patients with secondary loss of response for all agents.^105^ To date, no other relevant organizations have published recommendations regarding TDM.

Despite differences between the guidelines, TDM for TNF inhibitors is increasingly embedded in clinical practice.^106^ The use of TDM to optimize dosing creates a challenge when TNF inhibitors are part of head-to-head trials, as their designs do not generally include this as an option.^107^ This might be a disadvantage for trials comparing TNF inhibitors with biologics that have different modes of action. For example, the VARSITY study compared adalimumab and vedolizumab, but treatment optimization through reactive dose adjustments was not possible due to the study design.^107,108^ It should, however, be noted that data from the SERENE-UC study suggest that use of TDM to guide adalimumab dosing may not have made any difference to outcomes from the VARSITY study.^102^ Another ongoing phase III study, comparing infliximab with etrolizumab in TNF inhibitor-naïve patients with UC (GARDENIA), does not offer the option for dose escalation in instances of treatment failure.^107,109^

## Management of TNF inhibitor failure

Although TNF inhibitors have transformed outcomes for many patients with IBD, it is estimated that 10–30% of patients will not respond to initial therapy (primary non-response) and 23–46% will lose response over time (secondary loss of response).^110^ Primary non-response and secondary loss of response can arise from three different scenarios: immunogenicity-mediated failure (in patients with low or undetectable trough concentrations and high titres of anti-drug antibodies), non-immunogenicity-mediated pharmacokinetic failure (in patients with subtherapeutic trough concentrations and absent anti-drug antibodies) and mechanistic or pharmacodynamic failure (in patients with therapeutic drug levels and absent anti-drug antibodies).^58,104^ As such, management approaches to treatment failure depend largely on drug concentrations and anti-drug antibody titres and have been reviewed in detail in other publications.^104^ Guidelines recommend evaluating patients who experience treatment failure with TNF inhibitors to determine whether their symptoms are the result of active disease.^53,58,97,98^ In addition, it is recommended that patients who have previously developed anti-drug antibodies receive combination therapy with an immunosuppressive agent.^58^

Currently, there are few clinical recommendations and/or guidelines on how to manage primary non-response to TNF inhibitor therapy, or which agent to move onto when patients have mechanistic primary non-response.^29^ Suggested approaches include surgery or swapping to a different drug class, with some guidance provided on the selection of alternative molecules.^54,60^ There is also some evidence suggesting that some primary non-responders can achieve clinical benefit by switching to an alternative TNF inhibitor.^111^

For patients with a secondary loss of response to a TNF inhibitor, guidelines recommend switching between alternative TNF inhibitors, swapping drug class, or surgery.^52,60^ Dose optimization with reactive TDM is also recommended by guidelines,^53,58,98^ but there is currently a lack of large robust studies supporting this.

A number of small studies have shown that switching between TNF inhibitors can be an efficacious treatment strategy in patients with secondary loss of response to a first TNF inhibitor.^112-114^ These observations are supported by the findings of the ENEIDA Registry, a large cohort study (*N* = 1122), which reported that 55% of patients who switched to a second TNF inhibitor following failure of or intolerance to a prior TNF inhibitor achieved remission in the short term, but a proportion of them went on to experience further loss of response (19% per patient-year).^114^Two systematic reviews also support switching to a second TNF inhibitor following treatment failure.^115,116^ A meta-analysis on the clinical success of switching to a second TNF inhibitor after the failure of a first one showed that the efficacy of second-line treatment in CD was dependent on the reason for discontinuing the first inhibitor; remission rates were higher when the reason for withdrawal was secondary loss of response *versus* primary non-response. Only six UC studies were identified that reported remission rates, ranging from 0% to 50%. However, it was not possible to estimate the pooled efficacy through a formal meta-analysis because of the heterogeneous study designs.^111^

Although not generally performed in clinical practice, as alternative drugs are now available, there is some observational evidence to suggest that a third TNF inhibitor may still be of benefit in patients who experience loss of response to two previous TNF inhibitors.^117^ Retrospective reviews of patients with IBD who failed two prior TNF inhibitors demonstrated that the use of a third TNF inhibitor was still effective^118-120^ and many patients continued to experience long-term benefit.^118,119^ The ENEIDA Registry study reported that 55% of the 71 patients who switched to a third TNF inhibitor achieved remission; the incidence of loss of response was 22% per patient-year.^114^ However, treatment duration for a specific TNF inhibitor appears to diminish with successive treatment cycling over time.^121^

Alternatively, swapping TNF inhibitor therapy for another class of drug has been recommended following treatment failure.^58^ Yet, current data demonstrate that patients who fail TNF inhibitor therapy do not respond well to alternative treatments.^29^ This may also be dependent on the therapy^29^ and reason for discontinuation of TNF inhibitors.^122^ A recent meta-analysis of eight IBD studies found that patients with primary non-response to TNF inhibitors were less likely to respond to non-TNF biologic therapies than those who discontinued due to secondary loss of response or intolerance.^123^

There are extremely limited data on the benefits of combination treatment following loss of response to TNF inhibitors. However, a recent prospective trial suggested that the addition of an immunosuppressive agent could provide more favourable clinical outcomes and pharmacokinetics than switching to TNF inhibitor monotherapy.^124^ Evidence is also accumulating that suggests that dual biologic therapy may offer a promising treatment option in patients who have failed multiple biologic treatments. A recent case series suggested that combining vedolizumab with TNF inhibitor therapy in patients with IBD is a promising treatment option in those experiencing loss of response to TNF inhibitors.^125,126^ This is further supported by an analysis of data from patients with refractory CD showing that patients experienced clinical, biomarker and endoscopic improvements following concomitant treatment with two biologics.^127^

Dose optimization with second-line options may help overcome pharmacokinetic causes of primary non-response but does not address the management of patients who are mechanistically resistant to TNF inhibitors. Therefore, it is important that the underlying reasons for primary non-response are established in order to inform treatment decisions. A recent study provided the first mechanistic insights into TNF treatment resistance in CD. Here, responders to TNF inhibitors displayed a higher expression of TNFR2 but not IL23R on T cells than non-responders. In non-responders, an upregulation of IL23R was observed on T cells during TNF inhibitor treatment, permitting a survival signal *via* IL-23. Thus, expansion of apoptosis-resistant intestinal TNFR2 + IL23R + T cells was associated with resistance to anti-TNF.^128^

## Patient profiling

**Figure 2.**
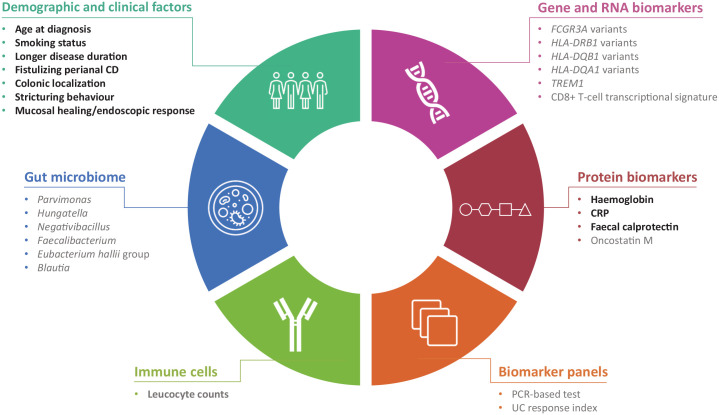
Current and potential prognostic and predictive biomarkers in IBD. Text highlighted in bold indicates biomarkers that are currently applicable in clinical practice; text in grey indicates potential future biomarkers. Data extracted from previous studies.^53,130-153^ CD, Crohn’s disease; CRP, C-reactive protein; IBD, inflammatory bowel disease; PCR, polymerase chain reaction; *TREM1*, Triggering Receptors Expressed on Myeloid cells 1; UC, ulcerative colitis.

There is growing interest in the use of prognostic and/or predictive biomarkers to support the selection of appropriate treatment strategies for patients with IBD. The presence of poor prognostic factors may indicate patients who are likely to benefit from early, more aggressive treatment. Moreover, identification of patients unlikely to respond to specific treatments would enable alternative treatment options to be considered at an earlier stage. Avoiding overtreatment of patients with a mild disease course is also critical to minimize safety risks and to avoid excessive costs to health care providers.^129^ Factors currently in use or under investigation for profiling patients are summarized in Figure 2.^53,130-153^

The risk of relapse is high after discontinuing TNF inhibitors,^130-133^ and clinical factors associated with an increased risk of relapse include younger age at diagnosis, smoking, longer disease duration and fistulizing perianal CD.^130^ Haemoglobin levels <145 g/L, high leucocyte count (>6 × 10^9^/L) and elevated CRP and faecal calprotectin are also associated with an increased risk of relapse.^130^ A retrospective study involving 1055 patients with IBD who discontinued TNF inhibitors demonstrated that treatment with adalimumab (*versus* infliximab), elective discontinuation of TNF inhibitors (*versus* discontinuation following a top-down strategy) and discontinuation due to adverse events (*versus* discontinuation following a top-down strategy) were all associated with a significantly increased risk of relapse in a multivariate analysis. Conversely, older age at the time of anti-TNF discontinuation and treatment with immunomodulators after anti-TNF discontinuation were associated with a significantly reduced risk of relapse.^132^ A separate sub-analysis for only patients with CD (*N* = 731) reported that the risk factors for relapse were the same as for the overall IBD patient population, with the addition of colonic localization and stricturing behaviour as factors associated with a higher risk of relapse.^132^ Retreatment with the same agent is effective in the majority of patients who relapse following treatment discontinuation.^130-132^ The ECCO–ESGAR guidelines recommend testing CRP and faecal calprotectin levels to monitor responsiveness to treatment.^98^ In addition, the ECCO–ESGAR guidelines highlight that elevated faecal calprotectin levels can be indicative of relapse, prior to the onset of clinical symptoms, and should therefore be used to monitor the condition of patients.^98^ The ACG guidelines also recommend faecal calprotectin as a non-invasive marker of relapse in patients with UC.^53^

Repeated exposure to TNF inhibitors can induce the formation of anti-drug antibodies, and the identification of patients at risk of immunogenicity could be used to guide treatment decisions.^134^ Specific variants in *FCGR3A*^135^ and *HLA-DRB1*^136^ have been associated with an increased risk of immunogenicity against TNF inhibitors, while other variants in *HLA-DQB1* and *HLA-DRB1* are thought to be protective.^136,137^ Recently, a study involving 1240 biologic-naïve patients from the observational PANTS (Personalising Anti-TNF Therapy in Crohn’s Disease Study) cohort reported that carriage of one or more of the *HLA-DQA1∗05* alleles almost doubles the risk of developing anti-drug antibodies to infliximab and adalimumab, independent of immunomodulator use.^134^ Of note, *HLA-DQA1∗05* is present in ~40% of the European population, and thus presents a challenge to the utility of *HLA* pretreatment testing for deciding whether to use combination therapy.

Up to 40% of patients with CD may be primary non-responders to TNF inhibitors,^117^ highlighting the clinical importance of identifying patients most likely to be responsive to anti-TNF therapy. As a result, numerous predictive biomarkers are under investigation (reviewed in previous studies^138-140^), including *TREM1* (Triggering Receptors Expressed on Myeloid cells 1) and the cytokines oncostatin M (OSM) and TNF. A meta-analysis of publicly available genome expression profiles from colon biopsies reported that the downregulation of *TREM1* in whole blood was associated with non-responsiveness to TNF inhibitors.^141^ Conversely, Verstockt *et al.* demonstrated that low expression of *TREM1* in whole blood and in mucosa at baseline was a biomarker for TNF-inhibitor-induced endoscopic remission in patients with IBD naïve to biologics.^142^ The discrepancy in the findings of these two studies may reflect the small patient populations included in both studies, differences in the definition of response and differences in the baseline characteristics of the patients, for example, ethnicity.^143^ High baseline levels of TNF production by CD14+ monocytes in peripheral blood of ⩾500 ρg/mL have recently been shown to be predictive of responsiveness to infliximab in patients with CD,^144^ while high pretreatment mucosal OSM expression was strongly predictive of primary non-response to TNF inhibitors in patients with IBD.^145^ Similar associations have since been reported between elevated levels of plasma OSM and poor response to infliximab in patients with CD.^146^ However, unlike *TREM1*, the mucosal OSM signal could not be translated into a blood/serological biomarker.^147^

The relationship between TNF inhibitor responsiveness and the gut microbiome and transcriptome has recently attracted increased interest. Dovrolis *et al.* identified that, at baseline, a high abundance of *Blautia, Faecalibacterium, Roseburia* and *Negativibacillus* genera in patients is associated with CD refractory to infliximab, whereas a high abundance of *Hungatella, Ruminococcus gnavus* and *Parvimonas* is indicative of responsiveness in patients. Moreover, this study also identified patterns of host inflammatory gene expression indicative of infliximab responsiveness. A subsequent correlation analysis demonstrated that the combined assessment of both the microbial taxa and host gene expression can help predict infliximab responsiveness in patients with CD.^148^

The ACG guidelines state that the achievement of mucosal healing is a major objective in the treatment of CD^58^ and is an emerging goal in the management of UC.^53^ Endoscopic response has been identified as a robust predictor of long-term mucosal healing in patients with CD receiving TNF inhibitors.^149^ Moreover, early mucosal healing was predictive of improved long-term outcomes in patients with UC receiving TNF inhibitors.^150^ Endoscopy is an essential tool for evaluating mucosal healing, but many patients are reluctant to undergo repeated procedures.^151^ Therefore, non-invasive surrogate markers of mucosal healing are being evaluated.^151^ For example, faecal calprotectin levels correlate with endoscopic activity.^98,151^ Recently, a UC response index using surrogate biomarkers (serum-neutrophil-related markers, CRP and neutrophil count) was shown to accurately detect mucosal healing in patients receiving TNF inhibitors.^152^

## Effects of TNF inhibitors on patients with intestinal stricture and postoperative recurrence after ileocaecal resection

Strictures are the most common complication in CD. In the STRIDENT trial, 77 patients with symptomatic CD strictures were assessed by imaging. Patients were randomized to intensive adalimumab treatment (160 mg weekly for 4 weeks, followed by 40 mg fortnightly maintenance plus thiopurine) or standard dose adalimumab monotherapy.^154^ The primary endpoint of improvement in the obstructive symptom score at 12 months was met by patients in both treatment groups, but the difference between groups was not significant (79% of intensive-treatment patients *versus* 64% of standard-dose patients; *p* = 0.17). Treatment failure was less common in the intensive-treatment group (10%) than in the standard-treatment group (28%) (*p* = 0.045), and improvement in stricture morphology was reported for a higher number of patients treated with intensive adalimumab (61%) compared with standard dose adalimumab (28%) (*p* = 0.009).^154^

Surgery can play a significant role in the management of IBD; however, postoperative recurrence of IBD has been observed in up to 73% of patients, with the presence of endoscopic lesions, although a smaller proportion of patients may exhibit symptoms.^155,156^ Randomized clinical studies have examined a variety of agents for the prevention of endoscopic and clinical postoperative recurrence, with guidelines recommending the use of imidazole antibiotics, thiopurines and TNF inhibitors as preventive therapies.^58^

The PREVENT study group evaluated the efficacy of infliximab *versus* placebo in preventing postoperative recurrence of IBD in a cohort of 297 patients worldwide.^157^ Randomization occurred within 45 days following ileocolonic resection with patients receiving infliximab (5 mg/kg) or placebo every 8 weeks for 200 weeks. Compared with placebo, a smaller proportion of patients treated with infliximab had clinical recurrence before or at week 76; however, this difference was not significant.^157^ A significantly smaller proportion of infinfliximab-treated patients had endoscopic recurrence when compared with those randomized to placebo (30.6% *versus* 60%, absolute risk reduction with infliximab 29.4%; 95% confidence interval: [18.6%–40.2%]; *p* < 0.001).^157^

A comparison of the efficacy of adalimumab and azathioprine noted a similar level of endoscopic recurrence, the primary study endpoint, for both treatment groups: 11/37 (29.7%) patients receiving adalimumab *versus* 8/24 (33.3%) patients receiving azathioprine (*p* = 0.76).^158^ These findings contrast with those of another study where endoscopic recurrence occurred in 6 of 28 (21%) adalimumab-treated patients compared with 33 of 73 (45%) of those treated with thiopurines (intention-to-treat population, *p* = 0.028), in patients meeting high-risk factors for postoperative recurrence.^159^

Real-world evidence from the ENEIDA Registry suggests that in clinical practice, TNF inhibitors are frequently used for the prevention of postoperative occurrence of IBD in patients experienced with such agents and who are often receiving concomitant immunosuppressants.^160^ Findings from this registry demonstrate that infliximab and adalimumab have similar efficacy for the prevention of postoperative recurrence, and that findings were consistent with results obtained in randomized controlled trials.^160^

## Potential impact of biosimilars

Due to its growing prevalence and advances in medical therapy and disease management, the impact of IBD on health care budgets is now considerable. In 2017, it was estimated that there were 6.8 million cases of IBD worldwide.^161^ Moreover, in 2013, the total direct costs of IBD in Europe were estimated to be as high as €5.6 billion per year.^162^ A major contributor to the economic burden of IBD is the high costs associated with the use of biologics, such as TNF inhibitors.^162^ In a recent pan-European, community-based inception cohort study (*N* = 1289) in IBD, expenditure on biologics accounted for 73% and 48% of costs in CD and UC, respectively, in the fifth year of follow-up. In addition, the overall mean yearly cost per patient-year for biologics was €866.^163^ The high costs of biologics have resulted in global access inequities, with patients in many countries not having access to these effective treatments.^164^ Cost and lack of reimbursement for biologics may also impact on the choice between earlier intervention, top-down and step-up treatment strategies.^165^

Biosimilars offer considerable cost savings compared with reference products and have the potential to expand overall use of biologics and improve health outcomes.^166^ Biosimilars receive regulatory approval on the basis of a robust evaluation process, including extensive *in vitro* analytical studies, pharmacodynamic evaluations and comparative clinical trials in healthy volunteers and sensitive patient populations.^167^ ECCO and ACG agree that approved biosimilar TNF inhibitors are as efficacious as the originator products, based on the rigorous regulatory pathways and postmarketing surveillance results.^53,168^ The first TNF biosimilar to be approved as a treatment for IBD was CT-P13 (Remsima^®^, Celltrion; Inflectra^®^, Pfizer), a biosimilar of infliximab.^169^ Although initially approved on the basis of extrapolation from clinical studies performed in rheumatic diseases, a recent phase III, randomized, controlled trial demonstrated that CT-P13 was non-inferior to reference infliximab in CD.^170^ In addition, NOR-SWITCH, a double-blind, non-inferiority, phase IV study demonstrated that switching from reference infliximab to CT-P13 was not inferior to continued treatment with the reference product. Although not powered to show non-inferiority in individual diseases, this study did include 155 patients with CD and 93 patients with UC.^171^ Additional biosimilars to infliximab (SB2 [Flixabi^®^, Samsung Biopesis] and PF-06438179/GP1111 [Zessly^®^, Sandoz]) and biosimilars to adalimumab (ABP 501 [Amgevita^®^, Amgen], BI 695501 [Cyltezo^®^, Boehringer Ingelheim], FKB327 [Hulio^®^, Mylan and Fujifilm Kyowa Kirin Biologics], GP2017 [Hyrimoz^®^, Sandoz], MSB11022^11^ [Idacio^®^, Fresenius Kabi], SB5 [Imraldi^®^, Biogen and Samsung Bioepis] and PF-06410293^172^ [Abrilada™, Amsparity^®^, Pfizer]) have subsequently been approved as treatments for IBD.^173^ A summary of biosimilar TNF inhibitors approved for IBD is presented in Figure 1.

## Future directions for TNF inhibitors

TNF inhibitors will continue to be relevant as a treatment for IBD over the next decade. Registry data demonstrate that TNF inhibitors have a better safety profile than steroids across immune-mediated inflammatory diseases, and the past two decades have seen a decrease in the use of immunomodulators and conventional therapies in favour of anti-TNF agents. In patients with steroid-refractory acute severe UC, infliximab remains an important second-line treatment option. TNF inhibitors also offer an effective treatment for the musculoskeletal, cutaneous and ocular EIMs that many patients with IBD develop over time and which can have a significant and detrimental effect on their quality of life. With the introduction of TNF inhibitor biosimilars, considerable cost savings are available, facilitating improved global access to, and use of, this important class of treatments. Potentially, this will lead to improved health outcomes in IBD and the continued popularity of TNF inhibitors as newer, but more expensive, treatment options emerge.

As the landscape of IBD treatment options widens, the potential opportunities for using novel combinations of TNF inhibitors with agents that target different immune pathways such as ustekinumab, vedolizumab, Janus kinase inhibitors or anti-P19 antibodies increase. As conventional delivery of TNF inhibitors can be problematic, new drug delivery systems that enable mucosal targeted delivery or sustained release are currently emerging. These may include enteric-coated TNF inhibitors^174^ or nanotechnology using nanocarriers, nanoparticles or polymeric nanoparticle systems.^175^

TNF inhibitors ushered in the biologic therapy era for IBD and are likely to remain first-line biologic treatment options for some time. They have robust clinical effects in both CD and UC, and the wealth of efficacy and safety data is unparalleled. Combined with the cost benefits associated with biosimilars, these are and will remain the cornerstone of IBD therapy.

## Supplemental Material

sj-docx-1-tag-10.1177_17562848211059954 – Supplemental material for Tumour necrosis factor inhibitors in inflammatory bowel disease: the story continues Supplemental material, sj-docx-1-tag-10.1177_17562848211059954 for Tumour necrosis factor inhibitors in inflammatory bowel disease: the story continues by Laurent Peyrin-Biroulet, William J. Sandborn, Remo Panaccione, Eugeni Domènech, Lieven Pouillon, Britta Siegmund, Silvio Danese and Subrata Ghosh in Therapeutic Advances in Gastroenterology

## References

[1] Windsor JW Kaplan GG. Evolving epidemiology of IBD. Curr Gastroenterol Rep 2019; 21: 40. 31338613 10.1007/s11894-019-0705-6

[2] Ananthakrishnan AN Kaplan GG Ng SC. Changing global epidemiology of inflammatory bowel diseases: sustaining health care delivery into the 21st century. Clin Gastroenterol Hepatol 2020; 18: 1252–1260. 32007542 10.1016/j.cgh.2020.01.028

[3] Kaplan GG. The global burden of IBD: from 2015 to 2025. Nat Rev Gastroenterol Hepatol 2015; 12: 720–727. 26323879 10.1038/nrgastro.2015.150

[4] Troncone E Marafini I Del Vecchio Blanco G , et al. Novel therapeutic options for people with ulcerative colitis: an update on recent developments with Janus kinase (JAK) inhibitors. Clin Exp Gastroenterol 2020; 13: 131–139. 32440190 10.2147/CEG.S208020 PMC7211304

[5] Levin AD Wildenberg ME van den Brink GR. Mechanism of action of anti-TNF therapy in inflammatory bowel disease. J Crohns Colitis 2016; 10: 989–997. 26896086 10.1093/ecco-jcc/jjw053

[6] Casellas F Robles V Borruel N , et al. Restoration of quality of life of patients with inflammatory bowel disease after one year with antiTNFalpha treatment. J Crohns Colitis 2012; 6: 881–886. 22398074 10.1016/j.crohns.2012.01.019

[7] Vogelaar L Spijker AV van der Woude CJ. The impact of biologics on health-related quality of life in patients with inflammatory bowel disease. Clin Exp Gastroenterol 2009; 2: 101–109. 21694833 10.2147/ceg.s4512 PMC3108643

[8] Ruder B Atreya R Becker C. Tumour necrosis factor alpha in intestinal homeostasis and gut related diseases. Int J Mol Sci 2019; 20: 1887. 10.3390/ijms20081887 PMC6515381 30995806

[9] Sandborn WJ Hanauer SB Katz S , et al. Etanercept for active Crohn’s disease: a randomized, double-blind, placebo-controlled trial. Gastroenterology 2001; 121: 1088–1094. 11677200 10.1053/gast.2001.28674

[10] Rutgeerts P Sandborn WJ Fedorak RN , et al. Onercept for moderate-to-severe Crohn’s disease: a randomized, double-blind, placebo-controlled trial. Clin Gastroenterol Hepatol 2006; 4: 888–893. 16797249 10.1016/j.cgh.2006.04.022

[11] European Medicines Agency (EMA). Idacio summary of product characteristics, https://www.ema.europa.eu/en/documents/product-information/idacio-epar-product-information_en.pdf (accessed 14 January 2021).

[12] European Medicines Agency (EMA). Remicade summary of product characteristics, https://www.ema.europa.eu/en/documents/product-information/remicade-epar-product-information_en.pdf (accessed 14 January 2021).

[13] European Medicines Agency (EMA). Humira summary of product characteristics, https://www.ema.europa.eu/en/documents/product-information/humira-epar-product-information_en.pdf (accessed 14 January 2021).

[14] US Food and Drug Administration (FDA). Cimzia prescribing information, https://www.cimzia.com/themes/custom/cimzia/docs/CIMZIA_full_prescribing_information.pdf (accessed 14 January 2021).

[15] European Medicines Agency (EMA). Simponi summary of product characteristics, https://www.ema.europa.eu/en/documents/product-information/simponi-epar-product-information_en.pdf (accessed 14 January 2021).

[16] European Medicines Agency (EMA). Simponi summary of opinion. https://www.ema.europa.eu/en/documents/smop/chmp-post-authorisation-summary-positive-opinion-simponi_en-0.pdf (accessed 14 January 2021).

[17] European Medicines Agency (EMA). Inflectra summary of product characteristics, https://www.ema.europa.eu/en/documents/product-information/inflectra-epar-product-information_en.pdf (accessed 14 January 2021).

[18] European Medicines Agency (EMA). Remsima summary of product characteristics, https://www.ema.europa.eu/en/documents/product-information/remsima-epar-product-information_en.pdf (accessed 14 January 2021).

[19] European Medicines Agency (EMA). Flixabi summary of product characteristics, https://www.ema.europa.eu/en/documents/product-information/flixabi-epar-product-information_en.pdf (accessed 14 January 2021).

[20] European Medicines Agency (EMA). Amgevita summary of product characteristics, https://www.ema.europa.eu/en/documents/product-information/amgevita-epar-product-information_en.pdf (accessed 14 January 2021).

[21] European Medicines Agency (EMA). Imraldi summary of product characteristics, https://www.ema.europa.eu/en/documents/product-information/imraldi-epar-product-information_en.pdf (accessed 14 January 2021).

[22] European Medicines Agency (EMA). Zessly summary of product characteristics, https://www.ema.europa.eu/en/documents/product-information/zessly-epar-product-information_en.pdf (accessed 14 January 2021).

[23] European Medicines Agency (EMA). Hyrimoz summary of product characteristics. https://www.ema.europa.eu/en/documents/product-information/hyrimoz-epar-product-information_en.pdf (accessed 14 January 2021).

[24] European Medicines Agency (EMA). Hefiya summary of product characteristics, https://www.ema.europa.eu/en/documents/product-information/hefiya-epar-product-information_en.pdf (accessed 14 January 2021).

[25] European Medicines Agency (EMA). Halimatoz summary of product characteristics, https://www.ema.europa.eu/en/documents/product-information/halimatoz-epar-product-information_en.pdf (accessed 14 January 2021).

[26] European Medicines Agency (EMA). Hulio summary of product characteristics, https://www.ema.europa.eu/en/documents/product-information/hulio-epar-product-information_en.pdf (accessed 14 January 2021).

[27] European Medicines Agency (EMA). Amsparity summary of product characteristics, https://www.ema.europa.eu/en/documents/product-information/amsparity-epar-product-information_en.pdf (accessed 14 January 2021).

[28] European Medicines Agency (EMA). Yuflyma summary of opinion, https://www.ema.europa.eu/en/documents/smop-initial/chmp-summary-positive-opinion-yuflyma_en.pdf (accessed 14 January 2021).

[29] Fine S Papamichael K Cheifetz AS. Etiology and management of lack or loss of response to anti-tumor necrosis factor therapy in patients with inflammatory bowel disease. Gastroenterol Hepatol 2019; 15: 656–665. PMC6935028 31892912

[30] Adegbola SO Sahnan K Warusavitarne J , et al. Anti-TNF therapy in Crohn’s disease. Int J Mol Sci 2018; 19: 2244. 30065229 10.3390/ijms19082244 PMC6121417

[31] Ashton JJ Mossotto E Ennis S , et al. Personalising medicine in inflammatory bowel disease-current and future perspectives. Transl Pediatr 2019; 8: 56–69. 30881899 10.21037/tp.2018.12.03 PMC6382508

[32] Flamant M Roblin X. Inflammatory bowel disease: towards a personalized medicine. Therap Adv Gastroenterol 2018; 11: 1756283X17745029. 10.1177/1756283X17745029 PMC5784543 29383027

[33] Shivaji UN Sharratt CL Thomas T , et al. Review article: managing the adverse events caused by anti-TNF therapy in inflammatory bowel disease. Aliment Pharmacol Ther 2019; 49: 664–680. 30735257 10.1111/apt.15097

[34] Holbrook J Lara-Reyna S Jarosz-Griffiths H , et al. Tumour necrosis factor signalling in health and disease. F1000Res 2019; 8: F1000. 10.12688/f1000research.17023.1 PMC6352924 30755793

[35] Neurath MF. Cytokines in inflammatory bowel disease. Nat Rev Immunol 2014; 14: 329–342. 24751956 10.1038/nri3661

[36] Holtmann MH Douni E Schütz M , et al. Tumor necrosis factor-receptor 2 is up-regulated on lamina propria T cells in Crohn’s disease and promotes experimental colitis in vivo. Eur J Immunol 2002; 32: 3142–3151. 12555659 10.1002/1521-4141(200211)32:11<3142::AID-IMMU3142>3.0.CO;2-4

[37] Perrier C de Hertogh G Cremer J , et al. Neutralization of membrane TNF, but not soluble TNF, is crucial for the treatment of experimental colitis. Inflamm Bowel Dis 2013; 19: 246–253. 22649027 10.1002/ibd.23023

[38] Behm B Bickston S. Tumor necrosis factor-alpha antibody for maintenance of remission in Crohn’s disease. Cochrane Database Syst Rev 2008; 1: CD006893. 10.1002/14651858.CD006893 18254120

[39] Kaymakcalan Z Sakorafas P Bose S , et al. Comparisons of affinities, avidities, and complement activation of adalimumab, infliximab, and etanercept in binding to soluble and membrane tumor necrosis factor. Clin Immunol 2009; 131: 308–316. 19188093 10.1016/j.clim.2009.01.002

[40] Nesbitt A Fossati G Bergin M , et al. Mechanism of action of certolizumab pegol (CDP870): in vitro comparison with other anti-tumor necrosis factor alpha agents. Inflamm Bowel Dis 2007; 13: 1323–1332. 17636564 10.1002/ibd.20225

[41] Atreya R Zimmer M Bartsch B , et al. Antibodies against tumor necrosis factor (TNF) induce T-cell apoptosis in patients with inflammatory bowel diseases via TNF receptor 2 and intestinal CD14⁺ macrophages. Gastroenterology 2011; 141: 2026–2038. 21875498 10.1053/j.gastro.2011.08.032

[42] Povoleri GAM Lalnunhlimi S Steel KJA , et al. Anti-TNF treatment negatively regulates human CD4(+) T-cell activation and maturation in vitro, but does not confer an anergic or suppressive phenotype. Eur J Immunol 2020; 50: 445–458. 31722123 10.1002/eji.201948190 PMC7079027

[43] Evans HG Roostalu U Walter GJ , et al. TNF-α blockade induces IL-10 expression in human CD4+ T cells. Nat Commun 2014; 5: 3199. 24492460 10.1038/ncomms4199 PMC3918582

[44] Roberts CA Durham LE Fleskens V , et al. TNF blockade maintains an IL-10(+) phenotype in human effector CD4(+) and CD8(+) T cells. Front Immunol 2017; 8: 157. 28261215 10.3389/fimmu.2017.00157 PMC5309392

[45] Vos AC Wildenberg ME Arijs I , et al. Regulatory macrophages induced by infliximab are involved in healing in vivo and in vitro. Inflamm Bowel Dis 2012; 18: 401–408. 21936028 10.1002/ibd.21818

[46] Van den Brande JM Braat H van den Brink GR , et al. Infliximab but not etanercept induces apoptosis in lamina propria T-lymphocytes from patients with Crohn’s disease. Gastroenterology 2003; 124: 1774–1785. 12806611 10.1016/s0016-5085(03)00382-2

[47] Hiller A Biedermann L Fournier N , et al. The appearance of joint manifestations in the Swiss inflammatory bowel disease cohort. PLoS ONE 2019; 14: e0211554. 31039159 10.1371/journal.pone.0211554 PMC6490952

[48] Vavricka SR Galván JA Dawson H , et al. Expression patterns of TNFα, MAdCAM1, and STAT3 in intestinal and skin manifestations of inflammatory bowel disease. J Crohns Colitis 2018; 12: 347–354. 29182760 10.1093/ecco-jcc/jjx158

[49] Peyrin-Biroulet L Van Assche G Gómez-Ulloa D , et al. Systematic review of tumor necrosis factor antagonists in extraintestinal manifestations in inflammatory bowel disease. Clin Gastroenterol Hepatol 2017; 15: 25–36. 27392760 10.1016/j.cgh.2016.06.025

[50] Steeland S Libert C Vandenbroucke RE. A new venue of TNF targeting. Int J Mol Sci 2018; 19: 1442. 29751683 10.3390/ijms19051442 PMC5983675

[51] Parameswaran N Patial S. Tumor necrosis factor-α signaling in macrophages. Crit Rev Eukaryot Gene Expr 2010; 20: 87–103. 21133840 10.1615/critreveukargeneexpr.v20.i2.10 PMC3066460

[52] Harbord M Eliakim R Bettenworth D , et al. Third European evidence-based consensus on diagnosis and management of ulcerative colitis. Part 2: current management. J Crohns Colitis 2017; 11: 769–784. 28513805 10.1093/ecco-jcc/jjx009

[53] Rubin DT Ananthakrishnan AN Siegel CA , et al. ACG clinical guideline: ulcerative colitis in adults. Am J Gastroenterol 2019; 114: 384–413. 30840605 10.14309/ajg.0000000000000152

[54] Feuerstein JD Isaacs KL Schneider Y , et al. AGA clinical practice guidelines on the management of moderate to severe ulcerative colitis. Gastroenterology 2020; 158: 1450–1461. 31945371 10.1053/j.gastro.2020.01.006 PMC7175923

[55] Turner D Ruemmele FM Orlanski-Meyer E , et al. Management of paediatric ulcerative colitis, part 1: ambulatory care-an evidence-based guideline from the European Crohn’s and Colitis Organization and European Society of Paediatric Gastroenterology, Hepatology and Nutrition. J Pediatr Gastroenterol Nutr 2018; 67: 257–291. 30044357 10.1097/MPG.0000000000002035

[56] Turner D Ruemmele FM Orlanski-Meyer E , et al. Management of paediatric ulcerative colitis, part 2: acute severe colitis-an evidence-based consensus guideline from the European Crohn’s and Colitis Organization and the European Society of Paediatric Gastroenterology, Hepatology and Nutrition. J Pediatr Gastroenterol Nutr 2018; 67: 292–310. 30044358 10.1097/MPG.0000000000002036

[57] Torres J Bonovas S Doherty G , et al. ECCO guidelines on therapeutics in Crohn’s disease: medical treatment. J Crohns Colitis 2020; 14: 4–22. 31711158 10.1093/ecco-jcc/jjz180

[58] Lichtenstein GR Loftus EV Isaacs KL , et al. ACG clinical guideline: management of Crohn’s disease in adults. Am J Gastroenterol 2018; 113: 481–517. 29610508 10.1038/ajg.2018.27

[59] Terdiman JP Gruss CB Heidelbaugh JJ , et al. American Gastroenterological Association Institute guideline on the use of thiopurines, methotrexate, and anti-TNF-alpha biologic drugs for the induction and maintenance of remission in inflammatory Crohn’s disease. Gastroenterology 2013; 145: 1459–1463. 24267474 10.1053/j.gastro.2013.10.047

[60] van Rheenen PF Aloi M Assa A , et al. The medical management of paediatric Crohn’s disease: an ECCO-ESPGHAN guideline update. J Crohns Colitis. Epub ahead of print 7 October 2020. DOI: 10.1093/ecco-jcc/jjaa161. 33026087

[61] Nguyen GC Loftus EV Jr Hirano I , et al. American Gastroenterological Association Institute guideline on the management of Crohn’s disease after surgical resection. Gastroenterology 2017; 152: 271–275. 27840074 10.1053/j.gastro.2016.10.038

[62] Hanauer SB Sandborn WJ Lichtenstein GR. Evolving considerations for thiopurine therapy for inflammatory bowel diseases-a clinical practice update: commentary. Gastroenterology 2019; 156: 36–42. 30195449 10.1053/j.gastro.2018.08.043

[63] Kotlyar DS Osterman MT Diamond RH , et al. A systematic review of factors that contribute to hepatosplenic T-cell lymphoma in patients with inflammatory bowel disease. Clin Gastroenterol Hepatol 2011; 9: 36–41. 20888436 10.1016/j.cgh.2010.09.016

[64] Toruner M Loftus EV Jr Harmsen WS , et al. Risk factors for opportunistic infections in patients with inflammatory bowel disease. Gastroenterology 2008; 134: 929–936. 18294633 10.1053/j.gastro.2008.01.012

[65] Boyapati RK Torres J Palmela C , et al. Withdrawal of immunosuppressant or biologic therapy for patients with quiescent Crohn’s disease. Cochrane Database Syst Rev 2018; 5: CD012540. 10.1002/14651858.CD012540.pub2 PMC6494506 29756637

[66] Van Assche G Magdelaine-Beuzelin C D’Haens G , et al. Withdrawal of immunosuppression in Crohn’s disease treated with scheduled infliximab maintenance: a randomized trial. Gastroenterology 2008; 134: 1861–1868. 18440315 10.1053/j.gastro.2008.03.004

[67] Drobne D Bossuyt P Breynaert C , et al. Withdrawal of immunomodulators after co-treatment does not reduce trough level of infliximab in patients with Crohn’s disease. Clin Gastroenterol Hepatol 2015; 13: 514–521.e4. 10.1016/j.cgh.2014.07.027 25066841

[68] Yerushalmy-Feler A Moran-Lev H Galai T , et al. Predictors for complicated disease course after stepping down from combination to antitumor necrosis factor alpha monotherapy in children with inflammatory bowel disease. Digestion 2020; 101: 121–128. 30625460 10.1159/000496273

[69] Roblin X Boschetti G Williet N , et al. Azathioprine dose reduction in inflammatory bowel disease patients on combination therapy: an open-label, prospective and randomised clinical trial. Aliment Pharmacol Ther 2017; 46: 142–149. 28449228 10.1111/apt.14106

[70] Pouillon L Lamoureux A Pineton de Chambrun G , et al. Dose de-escalation to adalimumab 40 mg every three weeks in patients with inflammatory bowel disease – a multicenter, retrospective, observational study. Dig Liver Dis 2019; 51: 236–241. 30502230 10.1016/j.dld.2018.10.022

[71] Papamichael K Karatzas P Mantzaris GJ. De-escalation of infliximab maintenance therapy from 8- to 10-week dosing interval based on faecal calprotectin in patients with Crohn’s disease. J Crohns Colitis 2016; 10: 371–372. 26546496 10.1093/ecco-jcc/jjv206 PMC4957471

[72] Van Steenbergen S Bian S Vermeire S , et al. Dose de-escalation to adalimumab 40 mg every 3 weeks in patients with Crohn’s disease – a nested case-control study. Aliment Pharmacol Ther 2017; 45: 923–932. 10.1111/apt.13964 28164321

[73] Torres P Cañete F Núñez L , et al. Spacing the administration interval of anti-TNF agents: a valid strategy for patients with inflammatory bowel disease? Dig Dis Sci 2020; 65: 2036–2043. 31858325 10.1007/s10620-019-06010-w

[74] Ungaro RC Aggarwal S Topaloglu O , et al. Systematic review and meta-analysis: efficacy and safety of early biologic treatment in adult and paediatric patients with Crohn’s disease. Aliment Pharmacol Ther 2020; 51: 831–842. 32202328 10.1111/apt.15685 PMC7160034

[75] Schreiber S Reinisch W Colombel JF , et al. Subgroup analysis of the placebo-controlled CHARM trial: increased remission rates through 3 years for adalimumab-treated patients with early Crohn’s disease. J Crohns Colitis 2013; 7: 213–221. 22704916 10.1016/j.crohns.2012.05.015

[76] Colombel JF Rutgeerts PJ Sandborn WJ , et al. Adalimumab induces deep remission in patients with Crohn’s disease. Clin Gastroenterol Hepatol 2014; 12: 414–422. 23856361 10.1016/j.cgh.2013.06.019

[77] Lunder AK Jahnsen J Bakstad LT , et al. Bowel damage in patients with long-term Crohn’s disease, assessed by magnetic resonance enterography and the Lémann Index. Clin Gastroenterol Hepatol 2018; 16: 75–82. 28694130 10.1016/j.cgh.2017.06.053

[78] Lauriot Dit Prevost C Azahaf M Nachury M , et al. Bowel damage and disability in Crohn’s disease: a prospective study in a tertiary referral centre of the Lémann Index and Inflammatory Bowel Disease Disability Index. Aliment Pharmacol Ther 2020; 51: 889–898. 10.1111/apt.15681 32221985

[79] Pariente B Mary JY Danese S , et al. Development of the Lémann Index to assess digestive tract damage in patients with Crohn’s disease. Gastroenterology 2015; 148: 52–63. 25241327 10.1053/j.gastro.2014.09.015

[80] Panchal H Wagner M Chatterji M , et al. Earlier anti-tumor necrosis factor therapy of Crohn’s disease correlates with slower progression of bowel damage. Dig Dis Sci 2019; 64: 3274–3283. 30607690 10.1007/s10620-018-5434-4 PMC7049096

[81] Kerur B Machan JT Shapiro JM , et al. Biologics delay progression of Crohn’s disease, but not early surgery, in children. Clin Gastroenterol Hepatol 2018; 16: 1467–1473. 29486253 10.1016/j.cgh.2018.02.027

[82] Kugathasan S Denson LA Walters TD , et al. Prediction of complicated disease course for children newly diagnosed with Crohn’s disease: a multicentre inception cohort study. Lancet 2017; 389: 1710–1718. 28259484 10.1016/S0140-6736(17)30317-3 PMC5719489

[83] Solberg IC Vatn MH Høie O , et al. Clinical course in Crohn’s disease: results of a Norwegian population-based ten-year follow-up study. Clin Gastroenterol Hepatol 2007; 5: 1430–1438. 18054751 10.1016/j.cgh.2007.09.002

[84] Gonczi L Bessissow T Lakatos PL. Disease monitoring strategies in inflammatory bowel diseases: what do we mean by ‘tight control’? World J Gastroenterol 2019; 25: 6172–6189. 31749591 10.3748/wjg.v25.i41.6172 PMC6848014

[85] D’Haens G Baert F van Assche G , et al. Early combined immunosuppression or conventional management in patients with newly diagnosed Crohn’s disease: an open randomised trial. Lancet 2008; 371: 660–667. 18295023 10.1016/S0140-6736(08)60304-9

[86] Hoekman DR Stibbe JA Baert FJ , et al. Long-term outcome of early combined immunosuppression versus conventional management in newly diagnosed Crohn’s disease. J Crohns Colitis 2018; 12: 517–524. 29401297 10.1093/ecco-jcc/jjy014

[87] Colombel JF Sandborn WJ Reinisch W , et al. Infliximab, azathioprine, or combination therapy for Crohn’s disease. N Engl J Med 2010; 362: 1383–1395. 20393175 10.1056/NEJMoa0904492

[88] Khanna R Bressler B Levesque BG , et al. Early combined immunosuppression for the management of Crohn’s disease (REACT): a cluster randomised controlled trial. Lancet 2015; 386: 1825–1834. 26342731 10.1016/S0140-6736(15)00068-9

[89] Parkes M Noor NM Dowling F , et al. PRedicting Outcomes For Crohn’s dIsease using a moLecular biomarkEr (PROFILE): protocol for a multicentre, randomised, biomarker-stratified trial. BMJ Open 2018; 8: e026767. 10.1136/bmjopen-2018-026767 PMC6286485 30523133

[90] Colombel J-F Panaccione R Bossuyt P , et al. Effect of tight control management on Crohn’s disease (CALM): a multicentre, randomised, controlled phase 3 trial. Lancet 2018; 390: 2779–2789. 10.1016/S0140-6736(17)32641-7 29096949

[91] Nuij V Fuhler GM Edel AJ , et al. Benefit of earlier anti-TNF treatment on IBD disease complications? J Crohns Colitis 2015; 9: 997–1003. 26223842 10.1093/ecco-jcc/jjv130

[92] Ma C Beilman CL Huang VW , et al. Similar clinical and surgical outcomes achieved with early compared to late anti-TNF induction in mild-to-moderate ulcerative colitis: a retrospective cohort study. Can J Gastroenterol Hepatol 2016; 2016: 2079582. 27478817 10.1155/2016/2079582 PMC4958475

[93] Bressler B Marshall JK Bernstein CN , et al. Clinical practice guidelines for the medical management of nonhospitalized ulcerative colitis: the Toronto consensus. Gastroenterology 2015; 148: 1035–1058. 25747596 10.1053/j.gastro.2015.03.001

[94] Steenholdt C. Proactive and reactive therapeutic drug monitoring of biologic therapies in inflammatory bowel disease are complementary, not mutually exclusive. Clin Gastroenterol Hepatol 2018; 16: 597–598. 29555230 10.1016/j.cgh.2017.11.033

[95] Franco DL Click B. Proactive versus reactive therapeutic drug monitoring of infliximab in Crohn’s disease: is the juice worth the squeeze? Inflamm Bowel Dis 2020; 26: 112–113. 31184363 10.1093/ibd/izz114

[96] Shah R Hoffman GR El-Dallal M , et al. Is therapeutic drug monitoring for anti-tumor necrosis factor agents in adults with inflammatory bowel disease ready for standard of care? A systematic review and meta-analysis. J Crohns Colitis 2020; 14: 1057–1065. 32064510 10.1093/ecco-jcc/jjaa029

[97] Feuerstein JD Nguyen GC Kupfer SS , et al. American Gastroenterological Association Institute guideline on therapeutic drug monitoring in inflammatory bowel disease. Gastroenterology 2017; 153: 827–834. 28780013 10.1053/j.gastro.2017.07.032

[98] Maaser C Sturm A Vavricka SR , et al. ECCO-ESGAR guideline for diagnostic assessment in IBD part 1: initial diagnosis, monitoring of known IBD, detection of complications. J Crohns Colitis 2019; 13: 144–164. 30137275 10.1093/ecco-jcc/jjy113

[99] Vande Casteele N Ferrante M Van Assche G , et al. Trough concentrations of infliximab guide dosing for patients with inflammatory bowel disease. Gastroenterology 2015; 148: 1320–1329. 10.1053/j.gastro.2015.02.031 25724455

[100] D’Haens G Vermeire S Lambrecht G , et al. Increasing infliximab dose based on symptoms, biomarkers, and serum drug concentrations does not increase clinical, endoscopic, and corticosteroid-free remission in patients with active luminal Crohn’s disease. Gastroenterology 2018; 154: 1343–1351.e1. 10.1053/j.gastro.2018.01.004 29317275

[101] Pouillon L Ferrante M Van Assche G , et al. Mucosal healing and long-term outcomes of patients with inflammatory bowel diseases receiving clinic-based *vs* trough concentration-based dosing of infliximab. Clin Gastroenterol Hepatol 2018; 16: 1276–1283. 29203225 10.1016/j.cgh.2017.11.046

[102] Colombel JF Panés J D’Haens G , et al. Higher vs. standard adalimumab maintenance regimens in patients with moderately to severely active ulcerative colitis: results from the SERENE-UC maintenance study. J Crohns Colitis 2020; 14: OP01.

[103] Assa A Matar M Turner D , et al. Proactive monitoring of adalimumab trough concentration associated with increased clinical remission in children with Crohn’s disease compared with reactive monitoring. Gastroenterology 2019; 157: 985–996. 31194979 10.1053/j.gastro.2019.06.003

[104] Mitrev N Vande Casteele N Seow CH , et al. Review article: consensus statements on therapeutic drug monitoring of anti-tumour necrosis factor therapy in inflammatory bowel diseases. Aliment Pharmacol Ther 2017; 46: 1037–1053. 29027257 10.1111/apt.14368

[105] Papamichael K Cheifetz AS Melmed GY , et al. Appropriate therapeutic drug monitoring of biologic agents for patients with inflammatory bowel diseases. Clin Gastroenterol Hepatol 2019; 17: 1655–1668. 30928454 10.1016/j.cgh.2019.03.037 PMC6661210

[106] Borg-Bartolo SP Boyapati RK Satsangi J , et al. Precision medicine in inflammatory bowel disease: concept, progress and challenges. F1000Res 2020; 9: F1000. 10.12688/f1000research.20928.1 PMC6993839 32047622

[107] Pouillon L Travis S Bossuyt P , et al. Head-to-head trials in inflammatory bowel disease: past, present and future. Nat Rev Gastroenterol Hepatol 2020; 17: 365–376. 32303700 10.1038/s41575-020-0293-9

[108] Sands BE Peyrin-Biroulet L Loftus EV , et al. Vedolizumab versus adalimumab for moderate-to-severe ulcerative colitis. N Engl J Med 2019; 381: 1215–1226. 31553834 10.1056/NEJMoa1905725

[109] US National Library of Medicine (ClinicalTrials.gov). A study comparing the efficacy and safety of etrolizumab to infliximab in participants with moderate to severe ulcerative colitis who are naïve to tumor necrosis factor (TNF) inhibitors (GARDENIA), https://clinicaltrials.gov/ct2/show/NCT02136069 (2014, accessed 10 July 2020).

[110] Roda G Jharap B Neeraj N , et al. Loss of response to anti-TNFs: definition, epidemiology, and management. Clin Transl Gastroenterol 2016; 7: e135. 26741065 10.1038/ctg.2015.63 PMC4737871

[111] Gisbert JP Marin AC McNicholl AG , et al. Systematic review with meta-analysis: the efficacy of a second anti-TNF in patients with inflammatory bowel disease whose previous anti-TNF treatment has failed. Aliment Pharmacol Ther 2015; 41: 613–623. 25652884 10.1111/apt.13083

[112] Sandborn WJ Rutgeerts P Enns R , et al. Adalimumab induction therapy for Crohn disease previously treated with infliximab: a randomized trial. Ann Intern Med 2007; 146 (Suppl 1): 829–838. 17470824 10.7326/0003-4819-146-12-200706190-00159

[113] Peeters H Louis E Baert F , et al. Efficacy of switching to infliximab in patients with Crohn’s disease with loss of response to adalimumab. Acta Gastroenterol Belg 2018; 81: 15–21. 29562373

[114] Casanova MJ Chaparro M Mínguez M , et al. Effectiveness and safety of the sequential use of a second and third anti-TNF agent in patients with inflammatory bowel disease: results from the ENEIDA Registry. Inflamm Bowel Dis 2020; 26: 606–616. 31504569 10.1093/ibd/izz192

[115] Ma C Panaccione R Heitman SJ , et al. Systematic review: the short-term and long-term efficacy of adalimumab following discontinuation of infliximab. Aliment Pharmacol Ther 2009; 30: 977–986. 19681810 10.1111/j.1365-2036.2009.04101.x

[116] Da W Zhu J Wang L , et al. Adalimumab for Crohn’s disease after infliximab treatment failure: a systematic review. Eur J Gastroenterol Hepatol 2013; 25: 885–891. 23817447 10.1097/MEG.0b013e32836220ab

[117] Ding NS Hart A De Cruz P. Systematic review: predicting and optimising response to anti-TNF therapy in Crohn’s disease – algorithm for practical management. Aliment Pharmacol Ther 2016; 43: 30–51. 26515897 10.1111/apt.13445

[118] Allez M Vermeire S Mozziconacci N , et al. The efficacy and safety of a third anti-TNF monoclonal antibody in Crohn’s disease after failure of two other anti-TNF antibodies. Aliment Pharmacol Ther 2010; 31: 92–101. 19709098 10.1111/j.1365-2036.2009.04130.x

[119] de Silva PS Nguyen DD Sauk J , et al. Long-term outcome of a third anti-TNF monoclonal antibody after the failure of two prior anti-TNFs in inflammatory bowel disease. Aliment Pharmacol Ther 2012; 36: 459–466. 22784296 10.1111/j.1365-2036.2012.05214.x

[120] Ferges W Rampertab SD Shafqet M , et al. Experience with anti-TNF-α biologic agents in succession in patients with Crohn’s disease: a retrospective analysis of a single center. J Clin Gastroenterol 2016; 50: 326–330. 25984976 10.1097/MCG.0000000000000338

[121] Hiroz P Vavricka SR Fournier N , et al. Analysis of TNF-antagonist switch over time and associated risk factors in the Swiss Inflammatory Bowel Disease Cohort. Scand J Gastroenterol 2014; 49: 1207–1218. 25120029 10.3109/00365521.2014.946082

[122] Kawalec P Moc’ko P Malinowska-Lipien I , et al. Efficacy and safety of ustekinumab in the induction therapy of TNF-α-refractory Crohn’s disease patients: a systematic review and meta-analysis. J Comp Eff Res 2017; 6: 601–612. 28660802 10.2217/cer-2017-0022

[123] Singh S George J Boland BS , et al. Primary non-response to tumor necrosis factor antagonists is associated with inferior response to second-line biologics in patients with inflammatory bowel diseases: a systematic review and meta-analysis. J Crohns Colitis 2018; 12: 635–643. 29370397 10.1093/ecco-jcc/jjy004 PMC7189966

[124] Roblin X Williet N Boschetti G , et al. Addition of azathioprine to the switch of anti-TNF in patients with IBD in clinical relapse with undetectable anti-TNF trough levels and antidrug antibodies: a prospective randomised trial. Gut 2020; 69: 1206–1212. 31980448 10.1136/gutjnl-2019-319758

[125] Mao EJ Lewin S Terdiman JP , et al. Safety of dual biological therapy in Crohn’s disease: a case series of vedolizumab in combination with other biologics. BMJ Open Gastroenterol 2018; 5: e000243. 10.1136/bmjgast-2018-000243 PMC6254738 30538822

[126] Buer LCT Høivik ML Warren DJ , et al. Combining anti-TNF-α and vedolizumab in the treatment of inflammatory bowel disease: a case series. Inflamm Bowel Dis 2018; 24: 997–1004. 29668901 10.1093/ibd/izx110

[127] Yang E Panaccione N Whitmire N , et al. Efficacy and safety of simultaneous treatment with two biologic medications in refractory Crohn’s disease. Aliment Pharmacol Ther 2020; 51: 1031–1038. 32329532 10.1111/apt.15719 PMC8032452

[128] Schmitt H Billmeier U Dieterich W , et al. Expansion of IL-23 receptor bearing TNFR2+ T cells is associated with molecular resistance to anti-TNF therapy in Crohn’s disease. Gut 2019; 68: 814–828. 29848778 10.1136/gutjnl-2017-315671 PMC6580782

[129] Chateau T Peyrin-Biroulet L. Evolving therapeutic goals in Crohn’s disease management. United European Gastroenterol J 2020; 8: 133–139. 10.1177/2050640619887316 PMC7079266 32213074

[130] Gisbert JP Marin AC Chaparro M. Systematic review: factors associated with relapse of inflammatory bowel disease after discontinuation of anti-TNF therapy. Aliment Pharmacol Ther 2015; 42: 391–405. 26075832 10.1111/apt.13276

[131] Bots SJ Kuin S Ponsioen CY , et al. Relapse rates and predictors for relapse in a real-life cohort of IBD patients after discontinuation of anti-TNF therapy. Scand J Gastroenterol 2019; 54: 281–288. 30907185 10.1080/00365521.2019.1582693

[132] Casanova MJ Chaparro M García-Sánchez V , et al. Evolution after anti-TNF discontinuation in patients with inflammatory bowel disease: a multicenter long-term follow-up study. Am J Gastroenterol 2017; 112: 120–131. 27958281 10.1038/ajg.2016.569

[133] Kennedy NA Heap GA Green HD , et al. Predictors of anti-TNF treatment failure in anti-TNF-naive patients with active luminal Crohn’s disease: a prospective, multicentre, cohort study. Lancet Gastroenterol Hepatol 2019; 4: 341–353. 30824404 10.1016/S2468-1253(19)30012-3

[134] Sazonovs A Kennedy NA Moutsianas L , et al. HLA-DQA1∗05 carriage associated with development of anti-drug antibodies to infliximab and adalimumab in patients with Crohn’s disease. Gastroenterology 2020; 158: 189–199. 31600487 10.1053/j.gastro.2019.09.041

[135] Romero-Cara P Torres-Moreno D Pedregosa J , et al. A FCGR3A polymorphism predicts anti-drug antibodies in chronic inflammatory bowel disease patients treated with anti-TNF. Int J Med Sci 2018; 15: 10–15. 29333082 10.7150/ijms.22812 PMC5765734

[136] Liu M Degner J Davis JW , et al. Identification of HLA-DRB1 association to adalimumab immunogenicity. PLoS ONE 2018; 13: e0195325. 29614084 10.1371/journal.pone.0195325 PMC5882140

[137] Atiqi S Hooijberg F Loeff FC , et al. Immunogenicity of TNF-inhibitors. Front Immunol 2020; 11: 312. 32174918 10.3389/fimmu.2020.00312 PMC7055461

[138] Stevens TW Matheeuwsen M Lönnkvist MH , et al. Systematic review: predictive biomarkers of therapeutic response in inflammatory bowel disease-personalised medicine in its infancy. Aliment Pharmacol Ther 2018; 48: 1213–1231. 30378142 10.1111/apt.15033

[139] Linares-Pineda TM Cañadas-Garre M Sánchez-Pozo A , et al. Pharmacogenetic biomarkers of response in Crohn’s disease. Pharmacogenomics J 2018; 18: 1–13. 28631723 10.1038/tpj.2017.27

[140] Gole B Potočnik U. Pre-treatment biomarkers of anti-tumour necrosis factor therapy response in Crohn’s disease-a systematic review and gene ontology analysis. Cells 2019; 8: 515. 31141991 10.3390/cells8060515 PMC6628089

[141] Gaujoux R Starosvetsky E Maimon N , et al. Cell-centred meta-analysis reveals baseline predictors of anti-TNFalpha non-response in biopsy and blood of patients with IBD. Gut 2019; 68: 604–614. 29618496 10.1136/gutjnl-2017-315494 PMC6580771

[142] Verstockt B Verstockt S Dehairs J , et al. Low TREM1 expression in whole blood predicts anti-TNF response in inflammatory bowel disease. eBioMedicine 2019; 40: 733–742. 30685385 10.1016/j.ebiom.2019.01.027 PMC6413341

[143] Verstockt B Verstockt S Blevi H , et al. TREM-1, the ideal predictive biomarker for endoscopic healing in anti-TNF-treated Crohn’s disease patients? Gut 2019; 68: 1531–1533. 30007919 10.1136/gutjnl-2018-316845

[144] Jessen B Rodriguez-Sillke Y Sonnenberg E , et al. Level of tumor necrosis factor production by stimulated blood mononuclear cells can be used to predict response of patients with inflammatory bowel diseases to infliximab. Clin Gastroenterol Hepatol 2021; 19: 721–731. 32272247 10.1016/j.cgh.2020.03.066

[145] West NR Hegazy AN Owens BMJ , et al. Oncostatin M drives intestinal inflammation and predicts response to tumor necrosis factor-neutralizing therapy in patients with inflammatory bowel disease. Nat Med 2017; 23: 579–589. 28368383 10.1038/nm.4307 PMC5420447

[146] Minar P Lehn C Tsai Y-T , et al. Elevated pretreatment plasma oncostatin M is associated with poor biochemical response to infliximab. Crohns Colitis 360 2019; 1: otz026. 10.1093/crocol/otz026 PMC6798793 31667468

[147] Verstockt S Verstockt B Vermeire S. Oncostatin M as a new diagnostic, prognostic and therapeutic target in inflammatory bowel disease (IBD). Expert Opin Ther Targets 2019; 23: 943–954. 31587593 10.1080/14728222.2019.1677608

[148] Dovrolis N Michalopoulos G Theodoropoulos GE , et al. The interplay between mucosal microbiota composition and host gene-expression is linked with infliximab response in inflammatory bowel diseases. Microorganisms 2020; 8: 438. 32244928 10.3390/microorganisms8030438 PMC7143962

[149] Alfaro I Masamunt MC Planell N , et al. Endoscopic response to tumor necrosis factor inhibitors predicts long term benefits in Crohn’s disease. World J Gastroenterol 2019; 25: 1764–1774. 31011260 10.3748/wjg.v25.i14.1764 PMC6465936

[150] Bossuyt P Baert F D’Heygere F , et al. Early mucosal healing predicts favorable outcomes in patients with moderate to severe ulcerative colitis treated with golimumab: data from the real-life BE-SMART cohort. Inflamm Bowel Dis 2019; 25: 156–162. 29920582 10.1093/ibd/izy219

[151] Daperno M Castiglione F de Ridder L , et al. Results of the 2nd part scientific workshop of the ECCO. II: Measures and markers of prediction to achieve, detect, and monitor intestinal healing in inflammatory bowel disease. J Crohns Colitis 2011; 5: 484–498. 10.1016/j.crohns.2011.07.003 21939926

[152] de Bruyn M Ringold R Martens E , et al. The ulcerative colitis response index for detection of mucosal healing in patients treated with anti-tumour necrosis factor. J Crohns Colitis 2019; 14: 176–184. 10.1093/ecco-jcc/jjz125 31628842

[153] Biasci D Lee JC Noor NM , et al. A blood-based prognostic biomarker in IBD. Gut 2019; 68: 1386–1395. 31030191 10.1136/gutjnl-2019-318343 PMC6691955

[154] Schulberg J Wright E Holt B , et al. Crohn’s disease strictures respond to drug treatment and treat-to-target intense combination therapy is more effective than standard anti-TNF therapy. The STRIDENT randomised controlled trial. ECCO 2021; P300.

[155] Rutgeerts P Geboes K Vantrappen G , et al. Predictability of the postoperative course of Crohn’s disease. Gastroenterology 1990; 99: 956–963. 2394349 10.1016/0016-5085(90)90613-6

[156] Pascua M Su C Lewis JD , et al. Meta-analysis: factors predicting postoperative recurrence with placebo therapy in patients with Crohn’s disease. Aliment Pharmacol Ther 2008; 28: 545–556. 18565159 10.1111/j.1365-2036.2008.03774.x

[157] Regueiro M Feagan BG Zou B , et al. Infliximab reduces endoscopic, but not clinical, recurrence of Crohn’s disease after ileocolonic resection. Gastroenterology 2016; 150: 1568–1578. 26946343 10.1053/j.gastro.2016.02.072

[158] López-Sanromán A Vera-Mendoza I Domènech E , et al. Adalimumab vs azathioprine in the prevention of postoperative Crohn’s disease recurrence. A GETECCU randomised trial. J Crohns Colitis 2017; 11: 1293–1301. 28402454 10.1093/ecco-jcc/jjx051

[159] De Cruz P Kamm MA Hamilton AL , et al. Efficacy of thiopurines and adalimumab in preventing Crohn’s disease recurrence in high-risk patients – a POCER study analysis. Aliment Pharmacol Ther 2015; 42: 867–879. 26314275 10.1111/apt.13353

[160] Cañete F Mañosa M Casanova MJ , et al. Adalimumab or infliximab for the prevention of early postoperative recurrence of Crohn disease: results from the ENEIDA Registry. Inflamm Bowel Dis 2019; 25: 1862–1870. 31006801 10.1093/ibd/izz084

[161] GBD 2017 Inflammatory Bowel Disease Collaborators. The global, regional, and national burden of inflammatory bowel disease in 195 countries and territories, 1990-2017: a systematic analysis for the Global Burden of Disease Study 2017. Lancet Gastroenterol Hepatol 2020; 5: 17–30. 31648971 10.1016/S2468-1253(19)30333-4 PMC7026709

[162] Burisch J Jess T Martinato M , et al. The burden of inflammatory bowel disease in Europe. J Crohns Colitis 2013; 7: 322–337. 23395397 10.1016/j.crohns.2013.01.010

[163] Burisch J Vardi H Schwartz D , et al. Health-care costs of inflammatory bowel disease in a pan-European, community-based, inception cohort during 5 years of follow-up: a population-based study. Lancet Gastroenterol Hepatol 2020; 5: 454–464. 32061322 10.1016/S2468-1253(20)30012-1

[164] Baumgart DC Misery L Naeyaert S , et al. Biological therapies in immune-mediated inflammatory diseases: can biosimilars reduce access inequities? Front Pharmacol 2019; 10: 279. 30983996 10.3389/fphar.2019.00279 PMC6447826

[165] Tsui JJ Huynh HQ. Is top-down therapy a more effective alternative to conventional step-up therapy for Crohn’s disease. Ann Gastroenterol 2018; 31: 413–424. 29991886 10.20524/aog.2018.0253 PMC6033752

[166] Gulacsi L Pentek M Rencz F , et al. Biosimilars for the management of inflammatory bowel diseases: economic considerations. Curr Med Chem 2019; 26: 259–269. 28393687 10.2174/0929867324666170406112304

[167] European Medicines Agency (EMA). Biosimilars in the EU, https://www.ema.europa.eu/en/documents/leaflet/biosimilars-eu-information-guide-healthcare-professionals_en.pdf (accessed 1 May 2020).

[168] Danese S Fiorino G Raine T , et al. ECCO position statement on the use of biosimilars for inflammatory bowel disease-an update. J Crohns Colitis 2017; 11: 26–34. 27927718 10.1093/ecco-jcc/jjw198

[169] Rudrapatna VA Velayos F. Biosimilars for the treatment of inflammatory bowel disease. Pract Gastroenterol 2019; 43: 84–91. 31435122 PMC6703165

[170] Ye BD Pesegova M Alexeeva O , et al. Efficacy and safety of biosimilar CT-P13 compared with originator infliximab in patients with active Crohn’s disease: an international, randomised, double-blind, phase 3 non-inferiority study. Lancet 2019; 393: 1699–1707. 30929895 10.1016/S0140-6736(18)32196-2

[171] Jørgensen KK Olsen IC Goll GL , et al. Switching from originator infliximab to biosimilar CT-P13 compared with maintained treatment with originator infliximab (NOR-SWITCH): a 52-week, randomised, double-blind, non-inferiority trial. Lancet 2017; 389: 2304–2316. 28502609 10.1016/S0140-6736(17)30068-5

[172] US Food and Drug Administration. Abrilada prescribing information, https://www.accessdata.fda.gov/drugsatfda_docs/label/2019/761118s000lbl.pdf (accessed 12 October 2020).

[173] Al Sulais E AlAmeel T . Biosimilars to antitumor necrosis factor agents in inflammatory bowel disease. Biologics 2020; 14: 1–11. 32021084 10.2147/BTT.S236433 PMC6966952

[174] Crowe JS Roberts KJ Carlton TM , et al. Oral delivery of the anti-tumor necrosis factor α domain antibody, V565, results in high intestinal and fecal concentrations with minimal systemic exposure in cynomolgus monkeys. Drug Dev Ind Pharm 2019; 45: 387–394. 30395728 10.1080/03639045.2018.1542708

[175] Giron F Pastó A Tasciotti E , et al. Nanotechnology in the treatment of inflammatory bowel disease. Inflamm Bowel Dis 2019; 25: 1871–1880. 31560054 10.1093/ibd/izz205

