# Supplementary material for: Tumour necrosis factor inhibitors in inflammatory bowel disease: the story continues
Source: Ther Adv Gastroenterol. 2021 Dec 9;14:17562848211059954. doi: 10.1177/17562848211059954 (PMC8669878; doi:10.1177/17562848211059954)
Supplement: sj-docx-1-tag-10.1177_17562848211059954 – Supplemental material for Tumour necrosis factor inhibitors in inflammatory bowel disease: the story continues [file sj-docx-1-tag-10.1177_17562848211059954.docx]

**Supplemental material for Tumour necrosis factor inhibitors in inflammatory bowel disease: the story continues**

**Supplementary Table 1.** Literature search strings

| **Search** | **Search strings** |
| --- | --- |
| #1 | inflammatory bowel disease OR IBD OR crohn OR ulcerative colitis (all fields) |
| #2 | tumour necrosis factor inhibitor OR TNFi OR anti-TNF OR adalimumab OR infliximab OR etanercept OR golimumab OR certolizumab (all fields) |
| #3 | Early treatment OR early intervention OR early anti-TNF OR delayed treatment OR delayed intervention OR delayed anti-TNF OR TDM or therapeutic drug monitoring OR dosing frequency OR dose frequency OR dose optimisation OR dose intensification OR biomarker* OR patient profile* (title/abstract only) OR predict* factor* OR biosimilar (title/abstract only) |
| #4 | (loss of response OR treatment failure) AND (switch* OR cycl*) (title/abstract only) |
| #5 | #3 OR #4 |
| #6 | #1 AND #2 AND #5 |
| Search limits | English language, 2018−2020 |

IBD, inflammatory bowel disease; TDM, therapeutic drug monitoring; TNF(i), tumour necrosis factor (inhibitor).

**Supplementary Table 2.** Key data from recent prospective and retrospective studies in Crohn's disease supporting early intervention with TNF inhibitors

| **Study** | **Design** | **Treatment** | ***N*** | **Outcomes** |
| --- | --- | --- | --- | --- |
| Choi et al.^1^ 2018 | Retrospective paediatric study | 1. Early combination treatment (within 1 month of diagnosis) 2. Conventional step-up treatment | 33 | - Significant increases in linear growth (*p*=0.026) and weight (*p*=0.031) 3 years after diagnosis with early intervention - Significant difference between subgroups of Tanner stages 1–2 (*p*=0.016) with early intervention |
| Ling et al.^2^ 2018 | Paediatric cohort study | 1. Accelerated step-up treatment (within 3 months of diagnosis) 2. Conventional step-up treatment (after failing conventional therapy ≥3 months) | 43 | - Sustained primary response was observed in 17/43 (40%) and associated with shorter time from diagnosis to the initiation of TNF inhibitor (5.4 *versus* 18.7 months; *p*=0.006) |
| Frei et al.^3^ 2019 | Prospective cohort study | 1. Early treatment (<24 months after diagnosis) 2. Late treatment (≥24 months after diagnosis) 3. No TNF inhibitor | 1592 | - Reduced risk of stenosis with early *versus* late treatment (*p*<0.001) - Osteoporosis and anaemia were observed less frequently with early treatment *versus* late treatment  (*p*<0.001 and *p*=0.0046, respectively) or no TNF inhibitor (*p*<0.001 for both) - Early treatment was associated with fewer consultations (*p*=0.017), hospital outpatient visits  (*p*=0.038) and a composite of medical visits  (*p*=0.001) - Fewer patients were unable to work following early treatment *versus* late or no treatment (3.6% *versus* 8.8% *versus* 3.7%; *p*=0.016) |
| Mastronardi et al.^4^ 2019 | Retrospective, case-series study | 1. Early treatment (<12 months after diagnosis) 2. Late treatment (≥12 months after diagnosis) | 157 | Early *versus* late treatment:   - Clinical remission:^a^ 66.25% *versus* 33.77%; *p*<0.001 - Mucosal healing:^b^ 53.75% *versus* 20.78%; *p*<0.001 - Dose escalation: 30% *versus* 66.23%; *p*<0.001 |
| Jung et al.^5^ 2020 | Retrospective, cohort study | 1. Early treatment (≤12 months after diagnosis) 2. Late treatment (>12 months after diagnosis) | 1207 | Late treatment was associated with increased risk of:   - Surgery: aHR, 1.64; 95% CI 1.05, 2.55 - ER visits: aHR, 1.38; 95% CI 0.99, 1.94 |

^a^Defined according to the Harvey-Bradshaw Index (score <5).

^b^Defined according to the Simple Endoscopic Score for Crohn’s Disease (score <2).

aHR, adjusted hazard ratio; CD, Crohn’s disease; CI, confidence interval; ER, emergency room; TNF, tumour necrosis factor.

**References**

1. Choi J, Kang B, Kim M-J, et al. Early infliximab yields superior long-term effects on linear growth in pediatric Crohn's disease patients. *Gut Liver* 2018; 12: 255–262.

2. Ling J, Buurman D, Ravikumara M, et al. Accelerated step-up infliximab use is associated with sustained primary response in pediatric Crohn's disease. *Dig Dis Sci* 2018; 63: 1003–1010.

3. Frei R, Fournier N, Zeitz J, et al. Early initiation of anti-TNF is associated with favourable long-term outcome in Crohn's disease: 10-year-follow-up data from the Swiss IBD cohort study. *J Crohns Colitis* 2019; 13: 1292–1301.

4. Mastronardi M, Curlo M, Cavalcanti E, et al. Administration timing is the best clinical outcome predictor for adalimumab administration in Crohn's disease. *Front Med* 2019; 6: 234.

5. Jung YS, Han M, Park S, et al. Impact of early anti-TNF use on clinical outcomes in Crohn's disease: a nationwide population-based study. *Korean J Intern Med* 2020; 35: 1104–1113.
